# Lapachol, a compound targeting pyrimidine metabolism, ameliorates experimental autoimmune arthritis

**DOI:** 10.1186/s13075-017-1236-x

**Published:** 2017-03-07

**Authors:** Raphael S. Peres, Gabriela B. Santos, Nerry T. Cecilio, Valquíria A. P. Jabor, Michael Niehues, Bruna G. S. Torres, Gabriela Buqui, Carlos H. T. P. Silva, Teresa Dalla Costa, Norberto P. Lopes, Maria C. Nonato, Fernando S. Ramalho, Paulo Louzada-Júnior, Thiago M. Cunha, Fernando Q. Cunha, Flavio S. Emery, Jose C. Alves-Filho

**Affiliations:** 1 Department of Pharmacology, Ribeirão Preto Medical School, Center of Research in Inflammatory Diseases (CRID), University of São Paulo, Avenida Bandeirantes, 3900, Ribeirão Preto, São Paulo CEP: 14049-900 Brazil; 2 Department of Pharmaceutical Sciences, Faculty of Pharmaceutical Sciences of Ribeirão Preto, University of São Paulo, Avenida do Café s/n, Ribeirão Preto, CEP: 14040-903 Brazil; 3 NPPNS, Department of Physics and Chemistry, Faculty of Pharmaceutical Sciences of Ribeirão Preto, University of São Paulo, Avenida do Café s/n, Ribeirão Preto, Brazil; 4 Pharmaceutical Sciences Graduate Program, Faculty of Pharmacy, Federal University of Rio Grande do Sul, Sarmento Leite 521, Porto Alegre, Brazil; 5 Department of Pathology, Ribeirão Preto Medical School, University of São Paulo, Avenida Bandeirantes 3900, Ribeirão Preto, Brazil; 6 Department of Internal Medicine, Ribeirão Preto Medical School, Center of Research in Inflammatory Diseases (CRID), University of São Paulo, Avenida Bandeirantes 3900, Ribeirão Preto, Brazil

**Keywords:** Rheumatoid arthritis, Lapachol, Inflammation, Collagen-induced arthritis, Dihydroorotate dehydrogenase, DMARDs, Pyrimidine metabolism

## Abstract

**Background:**

The inhibition of pyrimidine biosynthesis by blocking the dihydroorotate dehydrogenase (DHODH) activity, the prime target of leflunomide (LEF), has been proven to be an effective strategy for rheumatoid arthritis (RA) treatment. However, a considerable proportion of RA patients are refractory to LEF. Here, we investigated lapachol (LAP), a natural naphthoquinone, as a potential DHODH inhibitor and addressed its immunosuppressive properties.

**Methods:**

Molecular flexible docking studies and bioactivity assays were performed to determine the ability of LAP to interact and inhibit DHODH. In vitro studies were conducted to assess the antiproliferative effect of LAP using isolated lymphocytes. Finally, collagen-induced arthritis (CIA) and antigen-induced arthritis (AIA) models were employed to address the anti-arthritic effects of LAP.

**Results:**

We found that LAP is a potent DHODH inhibitor which had a remarkable ability to inhibit both human and murine lymphocyte proliferation in vitro. Importantly, uridine supplementation abrogated the antiproliferative effect of LAP, supporting that the pyrimidine metabolic pathway is the target of LAP. In vivo, LAP treatment markedly reduced CIA and AIA progression as evidenced by the reduction in clinical score, articular tissue damage, and inflammation.

**Conclusions:**

Our findings propose a binding model of interaction and support the ability of LAP to inhibit DHODH, decreasing lymphocyte proliferation and attenuating the severity of experimental autoimmune arthritis. Therefore, LAP could be considered as a potential immunosuppressive lead candidate with potential therapeutic implications for RA.

**Electronic supplementary material:**

The online version of this article (doi:10.1186/s13075-017-1236-x) contains supplementary material, which is available to authorized users.

## Background

Rheumatoid arthritis (RA) is an autoimmune disease that is characterized by chronic articular inflammation with progressive joint destruction [1]. The current first-line therapy for RA patients includes the use of conventional disease-modifying antirheumatic drugs (DMARDs), such as methotrexate or leflunomide (LEF), in combination with short-term glucocorticoids. Moreover, the use of biological agents is employed as an alternative therapy in patients whose disease failed to respond to conventional DMARDs [2].

LEF is an isoxazole derivative with a potent immunosuppressive activity that was approved for the treatment of RA in 1998 [3]. LEF is a prodrug that is converted in vivo to its primary active metabolite A771726 (also known as teriflunomide). LEF blocks lymphocyte proliferation and hence the clonal expansion of autoreactive T cells in RA patients by inhibiting dihydroorotate dehydrogenase (DHODH), the mitochondrial rate-limiting enzyme in the de novo synthesis of pyrimidine ribonucleotides [4, 5]. The use of LEF is normally reserved for RA patients whose disease failed to respond to first-line DMARDs, before the introduction of biological DMARDs [6]. Nevertheless, despite it having demonstrated safety and efficacy, a substantial proportion of patients (around 30–40%) do not have an appropriate response to LEF [7]. Therefore, it is highly desirable to discover novel DHODH inhibitors as lead compounds for the development of new DMARD candidates.

Lapachol (LAP; 2-hydroxy-3-(3methyl-2-butenyl)-1,4-naphthoquinone) is a nonpolar naturally occurring naphthoquinone found in some Brazilian medicinal plants [8]. LAP and others naphthoquinones have been described as having a range of biological actions, including microbicidal, anti-inflammatory and antiproliferative activities [9–15]. In fact, it was demonstrated that LAP has potent antitumoral activity which was characterized by its ability to inhibit DNA and RNA synthesis in neoplastic cells [16]. Moreover, it was reported that LAP can reduce proliferation of the human keratinocytes in vitro, suggesting that it has potential antipsoriatic effects [17]. Despite the molecular mechanisms associated with these effects remaining poorly elucidated, it has been described that LAP and other naphthoquinones derivatives, such as lawsone and atovaquone, can inhibit DHODH activity [9]. However, the biological relevance of this effect was poorly characterized. In the present study, we investigated the potential immunosuppressive properties of LAP.

## Methods

### Preparation of LAP sodium salt

To a solution of LAP (500 mg, 2.06 mmol) in ethanol (20 ml) was added NaOH (112 mg, 0.28 mmol) and the reaction mixture was stirred for 24 h. After consumption of LAP, the reaction mixture was concentrated under reduced pressure and the solid residue was washed with dichloromethane (4×) and petroleum ether (4×) to afford a purple solid of 518 mg, 95% yield (^1^H NMR (300 MHz, D_2_O_d6_) δ7.66 (br s, 1H), 7.64 (br s, 1H), 7.53 (*br* t, *J* = 9 Hz, 1H), 7.41 (*br* t, *J* = 9Hz, 1H), 5.15 (*br* t, *J* = 9Hz, 1H), (3.06, d, *J* = 6Hz, 2H), 1.72 (s, 3H), 1.63 (s, 3H); ^13^C NMR (101 MHz, DMSO_d6_) δ 187.2, 178.4, 169.6, 136.0, 133.2, 131.3, 129.7, 127.5, 125.76 124.81, 124.4, 117.9, 25.59, 20.8, 14.1; HRMS-ESI m/z cald for: [M + Na]^+^ = 265.0835; found = 265.0834).

### Molecular modeling and docking procedures

We used nine human DHODH high-resolution crystal structures in complex with the following inhibitors: DHO1B0033 (PDB id: 4LS0); DSM338 (PDB id: 4OQV); O57 (PDB id: 4JS3); a brequinar analogue (PDB id: 4JTU); 221290 (PDB ID: 2WV8); amino-benzoic acid inhibitor 715 (PDB id: 3KVL); LEF derivative inhibitor 1 (PDB id: 3F1Q); another brequinar analogue (PDB id: 2B0M); and antiproliferative agent A771726 (PDB id: 1D3H). The crystal structure of hDHODH in complex with antiproliferative agent A771726 (PDB id: 1D3H) has been considered for flexible docking with A771726 and LAP, and the calculations were carried out using the GOLD (Genetic Optimisation for Ligand Docking) 5.2 software [18]. GOLD was comprehensively validated, reliably identifying the correct binding mode for a large range of test set cases, in a vast set of independent studies, with a rate of success in 70–80% of the PDB protein-ligand structures thus analyzed, such as reported in the literature [18, 19].

Here, a parameter set including a population of 100 conformers, 100,000 operations, 95 mutations, and 95 crossovers has been used. The simulations were then performed inside a selected region of the active site (sphere of 8.5 Å radius centered at *x* = 49.65, *y* = 42.13, *z* = –1.54), keeping the Leu46 side chain flexible (using a rotamers library). The number of docking simulations to be performed with each inhibitor was specified under 10 GA (genetic algorithm) runs, once each docking run can evolve to different ligand poses (pose = conformation + orientation). Thus, ten poses of highest score (top-ranked GOLD solutions) obtained for each compound were selected by using the CHEMPLP score function. In this case, a Piecewise Linear Potential (fPLP) is used to model the steric complementarity between protein and ligand, and for CHEMPLP the distance- and angle-dependent hydrogen and metal bonding terms from other fitness function also implemented in GOLD, so called ChemScore, are considered. CHEMPLP has been found to give the highest success rates for docking pose prediction as well as virtual screening experiments against diverse validation test sets and it was here chosen as the fitness function. Based on this CHEMPLP function, GOLD classifies the orientations of the molecules by a decreasing order of affinity (scores) with the binding site of the receptor [19].

Previous to the docking calculations and after the removal of the ligand as well as crystallographic waters of the hDHODH/A771726 complex structure, hydrogen atoms of the residues side chains were added and oriented in the active site region. Also, suitable 3D structures of the inhibitors A771726 and LAP were previously built and optimized with molecular mechanics (MMFF force field), followed by Hartree-Fock/Density Functional methods (full optimization at B3LYP/6-31G* level of calculation), using the Spartan’06 software.

### Pharmacokinetics study design

For administration to Wistar rats, LAP was dissolved in DMSO:Tween 80:glucose 5% in a proportion of 15:5:80 (v/v/v), resulting in a solution of 1 mg/ml (for intravenous (i.v.) administration) and another of 5 mg/ml (for oral administration). LAP was administered to rats as an i.v. bolus dose at 2 mg/kg (*n* = 7) and at two oral doses of 10 (*n* = 8) and 25 mg/kg (*n* = 6). LAP salt was administered as i.v. (2 mg/kg, *n* = 6) and oral doses (30 mg/kg equivalent to 27.5 mg/kg of LAP, *n* = 8). The i.v. doses were injected into the lateral tail vein, and oral doses were given by gavage. The doses were chosen based on previous toxicological and pharmacodynamic studies [20]. At predetermined time points (30 min before dosing and at 0.08, 0.25, 0.5, 1, 2, 4, 6, 8, 12, and 24 h) after LAP i.v. administration, blood samples (200–250 μl) were withdrawn into heparinized tubes via puncture of the lateral tail vein, opposite to the vein used for drug dosing. The same procedure was carried out after oral administration of LAP, with blood sampling at 0.25, 0.5, 1, 1.5, 2, 3, 6, 12, 24, and 30 h. After LAP sodium salt i.v. and oral dosing, blood samples were harvested up to 10 and 12 h, respectively. Plasma was obtained by centrifugation of blood samples (6800 × g, at 4 °C for 10 min) and stored at –80 °C until analysis by UPLC-MS/MS.

### Pharmacokinetic analysis

LAP and LAP sodium salt pharmacokinetic parameters after i.v. and oral administration were determined from individual plasma profiles by a noncompartmental approach (NCA). The peak plasma concentration (Cmax) and the time of maximum concentration (Tmax) were obtained by visual inspection of the data from the plasma concentration–time curve after oral dosing. Pharmacokinetic parameters such an elimination rate constant (λ), area under the curve (AUC0–∞), clearance (CLtot), half-life (t1/2), volume of distribution (Vdss), mean residence time (MRT), and bioavailability (Fabs) were determined using classical equations. The compartmental analyses were performed using SCIENTIST v.2.0.1 software (MicroMath®, USA). One- and two-compartment models with or without weighting schemes were evaluated. The best model to fit the data was chosen based on the random distribution of residuals, the correlation coefficient, and the model selection criterion (MSC) given by the software.

The individual plasma profiles of LAP and LAP sodium salt after i.v. administration were best described by a two-compartmental open model. Plasma profiles after oral administration with two different doses of LAP and one dose of LAP sodium salt were best described by the one-compartmental model.

### Plasma analysis by UPLC-MS/MS

LAP concentration in plasma samples was determined by a validated (FDA, US Food and Drug Administration, 2001) UPLC-MS/MS method [21]. Analyses were run on an Acquity UPLC BEH (Waters Acquity™) C18 column (2.1 × 50 mm, 1.7 μm particle size), with a flow of 300 μl/min at 35 °C. A gradient constituted of water (A) and acetonitrile (B) acidified with 0.1% acetic acid was used as follows: 0 min (90% A), 1 min (75% A), 7 min (50% A), 8.5 min (0% A) and 9.5 min (100% A). For the triple quadrupole, MS parameters were set as follows: capillary voltage (2.20 kV); extractor (3.0 V) source temperature (150 °C), desolvation temperature (300 °C), cone gas flow (50 l/h), and desolvation gas flow (700 l/h). For quantification, a multiple reaction monitoring method (MRM) was applied. For LAP, the transition of m/z 243 > 187 using cone energy of 24 V and collision energy of 19 V was determined as most appropriate for quantification (Calibration curves between 1 and 20,000 ng/ml of LAP, *R* > 0.99, low quantification limit of 1 ng/ml, and detection limit of 0.1 ng/ml).

### Sample preparation for pharmacokinetics studies

A total of 200 μl cold acetonitrile containing internal standard (2-methyl-amino-lapachol) at 5 μg/ml and 0.05% trifluoroacetic acid was added to 100 μl of plasma and vortexed for 20 s. Precipitated protein was removed by centrifugation (6800 × g at 4 °C for 10 min). A total of 200 μl of the supernatant was diluted with purified water 1:1 and filtered by a 0.22-μm membrane before analysis. To prepare the calibration curves, blank plasma samples were spiked with LAP and further processed as indicated. Animal samples with concentrations at the higher upper limit of the calibration curve were diluted with blank plasma before processing.

### Enzymatic assay

hDHODH activity was assessed using a colorimetric continuous assay that monitors 2,6-dichloroindophenol (DCIP) reduction. Change in absorbance at 610 nm was monitored over a period of 60 s at 25 °C using a microplate reader (Molecular Devices, SpectraMax 384 Plus, California, USA). The enzymatic reaction was analyzed in a total volume of 195 μl containing 50 mmol/l Tris, pH 8.15, 150 mmol/l KCl, 0.1% Triton X-100, 1 mmol/l l-dihydroorotate, 100 μmol/l CoQ0, and 60 μmol/l DCIP. The assay was started with 5 μl of 0.8 μmol/l stock of enzyme prepared in 50 mmol/l HEPES, pH 7.7, 400 mmol/l NaCl, 10% glycerol, 0.05% Thesit, and 1 mmol/l EDTA in a final concentration of enzyme at 20 nmol/l. A reference measurement was obtained by preparing the same solution without enzyme.

LAP was analyzed in quadruplicate for each concentration used. LAP sodium salt was prepared as a 10 mmol/l stock in DMSO. From this solution, dilutions were prepared in the assay mixture to achieve the range of 100 μmol/l to 0.35 nmol/l. Control enzyme activity in the absence of inhibitor was taken as 100%. The percentage of activity versus log of LAP concentration graph was drawn. The half maximal inhibitory concentration (IC_50_) values were calculated using a nonlinear fitting of the concentration–response data to the equation:

$$ activity\ \left(\%\right)= Bottom+\left[\frac{Top- Bottom}{10^{\log \left[ I\right]- \log \left[ I{C}_{50}\right]}+1}\right] $$

### Animals

Collagen-induced arthritis (CIA) and antigen-induced arthritis (AIA) models were carried out in male DBA1/J mice (10–12 weeks old) and male C57BL/6 mice (6 weeks old), respectively. The mice were bred and housed in the animal facility of the Ribeirão Preto Medical School (FMRP) at University of São Paulo. For the pharmacokinetic studies, male Wistar rats (200–300 g) were purchased from the State Foundation for the Research and Production in Health (FEPPS, Porto Alegre, Brazil). Animals received water and food ad libitum. All protocols were conducted in accordance with ethical guidelines and approved by the Animal Welfare Committee of FMRP and the Federal University of Rio Grande do Sul (Protocols: 53/2013 and 20244, respectively).

### Isolation of CD4 T cells

Human CD4 T cells were purified from the whole blood of healthy volunteers. Briefly, peripheral blood mononuclear cells (PBMCs) were isolated by Percoll gradient (Sigma-Aldrich, St. Louis, MO, USA). CD4 T cells were isolated from PBMCs using Isolation Kit (Miltenyi Biotec, Bergisch Gladbach, Germany). For murine CD4 T cells, lymph nodes from naive C57BL/6 male mice were harvested, and CD4 T cells were purified using Isolation Kit (Miltenyi Biotec, Bergisch Gladbach, Germany) according to the manufacturer’s recommendations.

### Proliferation assay

A total of 1 × 10^5^ CD4 T cells were labeled with 1 μM Dye Efluor® 670 (eBioscience, San Diego, CA, USA) for 15 min at 37 °C and cultured in RPMI-1640 10% FBS for 4 days in a 96-well U-bottom plate (Falcon, Franklin Lakes, New Jersey, USA) with the LAP salt diluted in RPMI-1640 (10, 30, and 100 μM), LEF diluted in RPMI-1640 (10, 30, and 100 μM; Arava®, Sanofi, France) and/or uridine (30, 100, and 300 μM; Sigma-Aldrich, St. Louis, MO, USA) in the presence of anti-CD3 (3 μg/ml) and anti-CD28 (1.5 μg/ml). The proliferation of CD4 T cells was determined by dye dilution in flow cytometry analysis. The results were expressed as the percentage of suppression using the following formula: [proliferation of CD4 T cells only – (proliferation of T CD4 cells with LEF or LAP)/proliferation of CD4 T cells only] × 100.

### Collagen-induced arthritis (CIA)

Male DBA/1 J mice were injected i.d. at the base of the tail with 200 μg bovine type II collagen (CII; a gift from Dr. David D. Brand, University of Tennessee Health Science Center) emulsified in Freund’s complete adjuvant (CFA) on day 0. Mice were boosted i.d. with CII (200 μg emulsified in Freund’s incomplete adjuvant (IFA)) on day 21. Mice were monitored daily for signs of arthritis. Scores were assigned based on erythema, swelling, or ankylosis present in each paw on a scale of 0 to 3, giving a maximum score of 12 per mouse. After arthritis induction, mice were treated orally with LAP (3 mg/kg and 10 mg/kg) or LEF (3 mg/kg) or saline daily. The clinical score was addressed every day after collagen boost. All mice were euthanized for histologic assessment of the hind limbs 4 weeks after the boost.

### Histological analysis

Femur-tibial joints were collected 4 weeks after CII boost, fixed in 4% (vol/vol) buffered formalin and decalcified in 10% EDTA for 2–3 weeks. The tissues were then trimmed, dehydrated in ethanol, and embedded in paraffin for the preparation of the slides. Histological assessment was carried out following routine staining. Joint sections were stained with hematoxylin and eosin (H&E) to analyze synovitis (inflammatory cell influx and synovial hyperplasia) or Safranin-O to visualize proteoglycan depletion and cartilage destruction. The severity of the joint damage was scored according to the criteria described by Wang et al. [22]: 0 = no destruction; 1 = minimal erosion; 2 = slight to moderate erosion in a limited area; 3 = more extensive erosion; 4 = general destruction. The degree of synovial pathology (i.e., synovitis) was scored using a scoring system that measured the thickness of the synovial cell layer on a scale of 0–3 (0 = 1–2 cells, 1 = 2–4 cells, 2 = 4–9 cells, and 3 = 10 or more cells) and cellular density in the synovial stroma on a scale of 0–3 (0 = normal cellularity, 1 = slightly increased cellularity, 2 = moderately increased cellularity, and 3 = greatly increased cellularity) [23].

### Cytokine quantification

Interleukin (IL)-17 and interferon (IFN)-γ cytokines were measured by enzyme-linked immunosorbent assay (ELISA) from hind paw homogenate of an individual mouse using antibodies according to the manufacturer’s instructions (R&D Systems, Minneapolis, MN, USA). The results were expressed as pg of cytokine/mg of tissue.

### Myeloperoxidase assay

Myeloperoxidase (MPO) activity in tissue homogenates was used as an index of neutrophil infiltration into paws from CIA mice as previously described [24].

### Measurement of liver enzymes

Serum concentrations of aspartate aminotransferase (AST) or alanine aminotransferase (ALT) were measured using commercial kits according to the manufacturer’s instructions (ID Labs Biotechnology Inc., London, Canada).

### Antigen-induced arthritis (AIA)

Mice were immunized with methylated bovine serum albumin (mBSA; Sigma-Aldrich, St. Louis, MO, USA) as described previously [25]. Briefly, mice were immunized with subcutaneous injection of an emulsion with mBSA (500 μg; Sigma-Aldrich, St. Louis, MO, USA) and CFA (2 mg/ml of inactivated *Mycobacterium tuberculosis*; Sigma-Aldrich, St. Louis, MO, USA). Booster injections of mBSA in IFA were given at 7 and 14 days after the first immunization. On day 21 after the first immunization, arthritis was induced by an intra-articular injection of mBSA (30 μg). During the AIA protocol, LAP (10 mg/kg) or saline (vehicle) was given orally every day from 12 to 21 days after first immunization.

### Determination of joint leucocyte infiltration

Leucocyte infiltration into the joints was assessed 6 h after intra-articular challenge with mBSA as previously described [26]. Briefly, articular infiltration of leukocytes was determined by washing the femur-tibial joint three times with 3.3 μl phosphate-buffered aline (PBS) + EDTA (0.2 M) and subsequent cell counting was performed in a Neubauer chamber. The results were expressed as the numbers of leucocyte × 10^4^ (mean ± SEM)/joint.

### Anti-mBSA antibody titer measurement

The titers of serum anti-mBSA antibody were measured by ELISA as previously described [26].

### Recall experiments

Cell suspension (1 × 10^5^ cells) of draining lymph nodes (inguinal) and spleen from naive and mBSA-immunized (treated or not with LAP) mice were stimulated in a 96-well round-bottom plate with mBSA (100 μg/ml) for 96 h. Next, the supernatant was collected to measure the levels of IL-17A, IFN-γ, and IL-4 by ELISA (R&D Systems, Minneapolis, MN, USA).

### Statistical analysis

Statistical analyses were performed using one-way nonparametric analysis of variance (ANOVA) followed by Bonferroni’s *t* test (for three or more groups) comparing all pairs of columns, or two-tailed Student’s *t* test (for two groups). *P* < 0.05 was considered statistically significant. Statistical analysis was performed with GraphPad Prism (GraphPad Software, San Diego, CA, USA).

## Results

### Synthesis, molecular modeling, and ability of LAP to inhibit DHODH activity

**Fig. 1:**
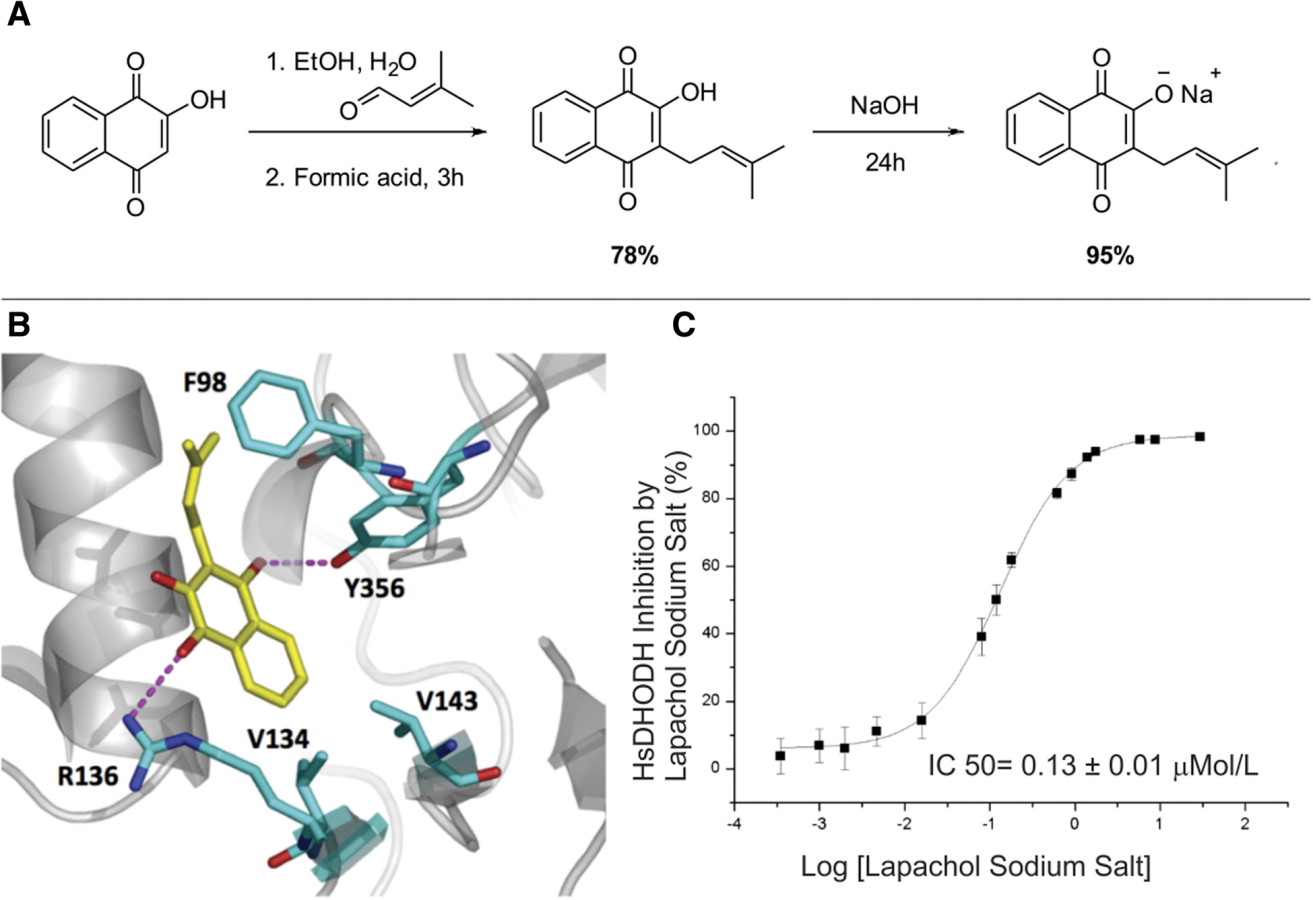
LAP is a potent inhibitor of DHODH activity. **a** Synthetic strategy for developing LAP sodium salt from lawsone. **b** The top-ranked GOLD solution for LAP is shown inside the hDHODH active site, in hydrophobic and polar contacts with selected residues of the enzyme. Hydrogen bonds are here represented by *dashed magenta lines*. **c** Inhibitory effect of LAP on human dihydroorotate dehydrogenase (*hDHODH*). Half maximal inhibitory concentration (*IC*_*50*_) determination of LAP based on DCIP colorimetric assay

LAP was synthetized using lawsone as the starting material in a one-pot methodology involving initial Knoevenagel condensation followed by formic acid catalyzed reduction in 78% yield. To increase the solubility of LAP, this naphthoquinone was treated with NaOH for 24 h to afford the LAP sodium salt in 95% yield (Fig. 1a). The structure of the salt was confirmed by ^1^H and ^13^C NMR and high-resolution mass spectrometry. Analysis of the LAP sodium salt stability on plasma and acid media (similar to the stomached conditions) was carried out using ultra-performance liquid-chromatography tandem mass spectrometry (UPLC-MS/MS) and it confirmed the instantaneous conversion of LAP sodium salt into a neutral molecule of LAP. The individual plasma profiles of LAP and LAP sodium salt after intravenous administration were identical and were best described by a two-compartmental open model, with a volume of distribution of 0.19 ± 0.03 l/kg, a total clearance of 0.04 ± 0.01 l/h/kg, and a half-life of 4.1 ± 1.1 h (Additional file 1: Figure S1; Additional file 2: Table S1). Linear pharmacokinetics were observed in the dose range investigated (2–25 mg/kg). Plasma profiles after oral administration of LAP and LAP sodium salt (at two different doses) were best described by the one-compartmental model, with bioavailabilities of 55–77% and 42%, respectively (Additional file 1: Figure S1; Additional file 3: Table S2).

Subsequently, we performed a computational analysis for the LAP molecule to propose a binding model in the active site of human DHODH (hDHODH), using a flexible docking approach implemented in the GOLD software [18]. As a control, we used the crystallographic binding mode of the active metabolite A771726 inside the hDHODH active site (PDB ID:1D3H). The binding model for LAP inside the hDHODH active site, proposed by docking, indicates that the hydrophobic pocket of hDHODH would be able to allow an accommodation of the prenyl (in hydrophobic contact with Phe98) and naphthoquinone moieties (in hydrophobic contact with Val134 and Val143) of LAP (Fig. 1b). Additional hydrogen bonds would be formed between the two carbonyl groups of such an inhibitor and Arg136 and Tyr356 of hDHODH, residues well conserved amongst the mammalian enzymes. The same polar contacts are also observed between these two hDHODH residues and the crystallographic inhibitor A771726 (PDB id: 1D3H) (Additional file 4: Figure S2) [5]. Next, to address whether LAP inhibits the DHODH activity, we carried out a cell-free DHODH activity assay by measuring the reduction of DCIP [12]. The hDHODH activity was significantly inhibited by LAP sodium salt with an IC_50_ value of 0.13 μM (Fig. 1c), indicating that LAP is a potent inhibitor of the hDHODH activity.

### LAP inhibits lymphocyte proliferation through inhibition of pyrimidine biosynthesis

**Fig. 2:**
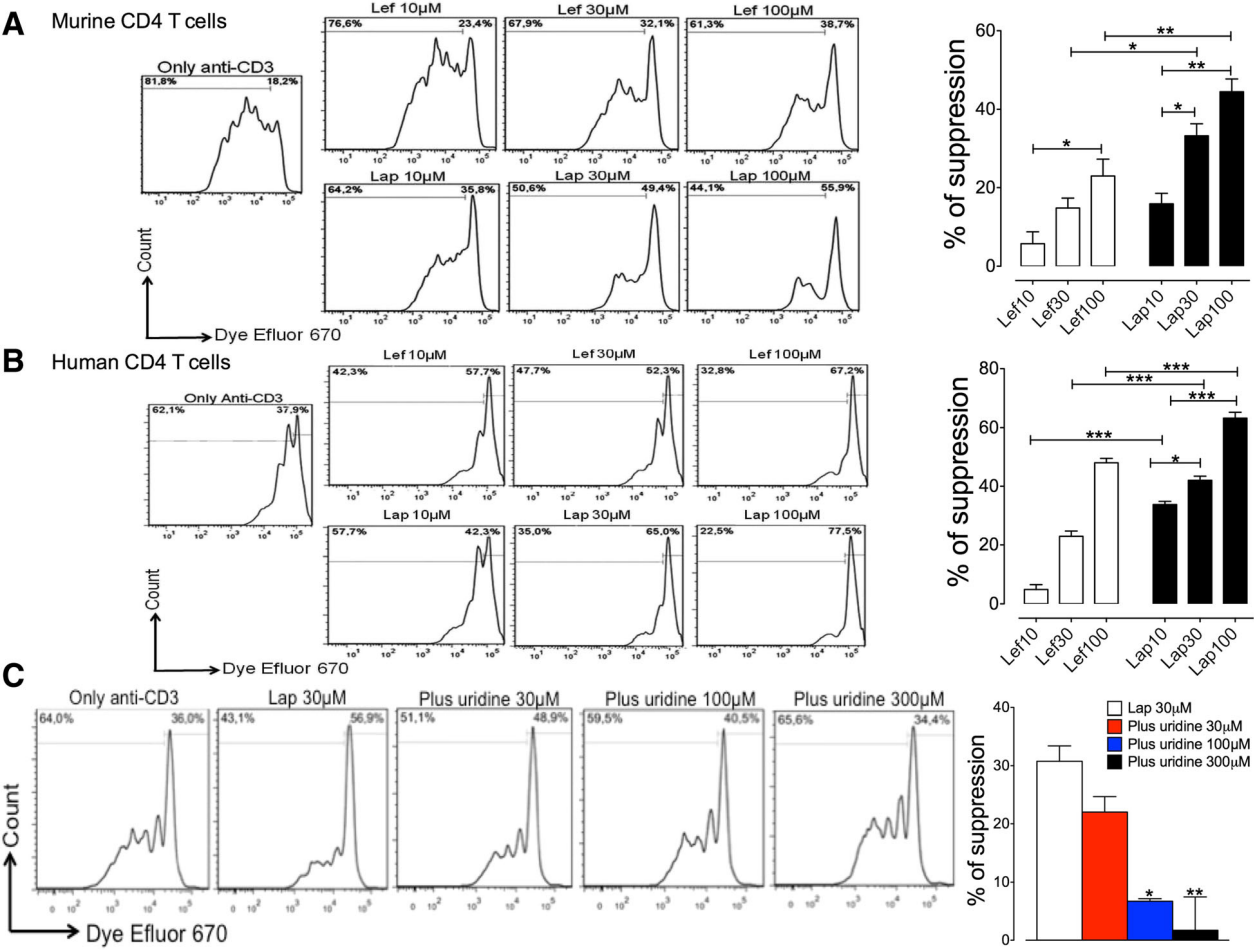
LAP modulates lymphocyte proliferation in a dependent pyrimidine biosynthesis manner. Murine CD4 T cells were purified from lymph nodes of naive C57BL/6 male mice and labeled with 1 μM Dye Efluor® 670 for 15 min at 37 °C and stimulated for 4 days in the presence of anti-CD3 (3 μg/ml) and anti-CD28 (1.5 μg/ml). Cells were concomitantly incubated in the presence of lapachol (*Lap*) or leflunomide (*Lef*) (10, 30 and 100 μM). **a** The percentage of suppression was assessed by the proliferation of murine CD4 T cells assessing dye dilution in flow cytometry analysis. Human CD4 T cells were purified from blood of healthy volunteers and labeled with 1 μM Dye Efluor® 670 for 15 min at 37 °C and stimulated for 4 days in the presence of anti-CD3 (3 μg/ml) and anti-CD28 (1.5 μg/ml). Cells were concomitantly incubated in the presence of LAP or LEF (10, 30 and 100 μM). **b** The percentage of suppression was assessed by the proliferation of human CD4 T cells assessing dye dilution in flow cytometry analysis. Human CD4 T cells were purified from blood of healthy volunteers and labeled with 1 μM Dye Efluor® 670 for 15 min at 37 °C and stimulated for 4 days in the presence of anti-CD3 (3 μg/ml) and anti-CD28 (1.5 μg/ml). Cells were concomitantly incubated or not with LAP (10, 30, and 100 μM) and/or uridine (30, 100, and 300 μM). **c** The percentage of suppression was assessed by the proliferation of human CD4 T cells assessing dye dilution in flow cytometry analysis. The results were expressed using the following formula: [proliferation of CD4 T cells only – (proliferation of CD4 T cells with LEF or LAP)/proliferation of CD4 T cells only] × 100. Data are shown as mean ± SEM, *n* = 5 per group. *P < 0.05, **P < 0.01, ***P < 0.001

We next assessed the antiproliferative effect of LAP. To this end, freshly isolated mouse CD4 T cells were labelled with Dye Efluor 670 and stimulated with anti-CD3/CD28 in the presence of LAP or LEF (10, 30, and 100 μM) for 4 days. As shown in Fig. 2a, LAP or LEF inhibited the proliferation of murine CD4 T cells in a dose-dependent manner. We also investigated the effect of LAP in human CD4 T cells isolated from the peripheral blood of healthy donors. Similar to that observed with murine cells, we also found a dose-dependent inhibition of human T-cell proliferation in the presence of LAP or LEF (Fig. 2b). However, we found that LAP exhibited a greater ability to suppress the proliferation of human and murine CD4 T cells than was observed with LEF at the same equivalent concentrations (Fig. 2a and b). Additionally, we performed annexin-V/propidium iodide (PI) staining of human CD4 T cells treated with LAP or LEF to assess apoptotic cell death. While LEF showed no toxic effects at all concentrations, flow cytometric analysis revealed that only the highest concentration of LAP (100 μM) was toxic (Additional file 5: Table S3). Thus, the reduction of T-cell proliferation by LAP below 100 μM was primarily due to inhibition of the proliferative response rather than a reduction of cellular viability by toxicity.

The antiproliferative effect of LEF is completely reversed by supplementation of uridine, supporting that DHODH is the target for LEF [4]. We then investigated whether the antiproliferative effect of LAP is also due to targeting DHODH. To this end, human CD4 T cells were pretreated with LAP in the presence of different concentrations of uridine. As shown in Fig. 2c, uridine was able to reverse the antiproliferative effect of LAP in a dose-dependent manner. Of note, uridine alone had a minimal effect on the proliferative response of anti-CD3-stimulated T cells.

### LAP reduces the severity of experimental arthritis

**Fig. 3:**
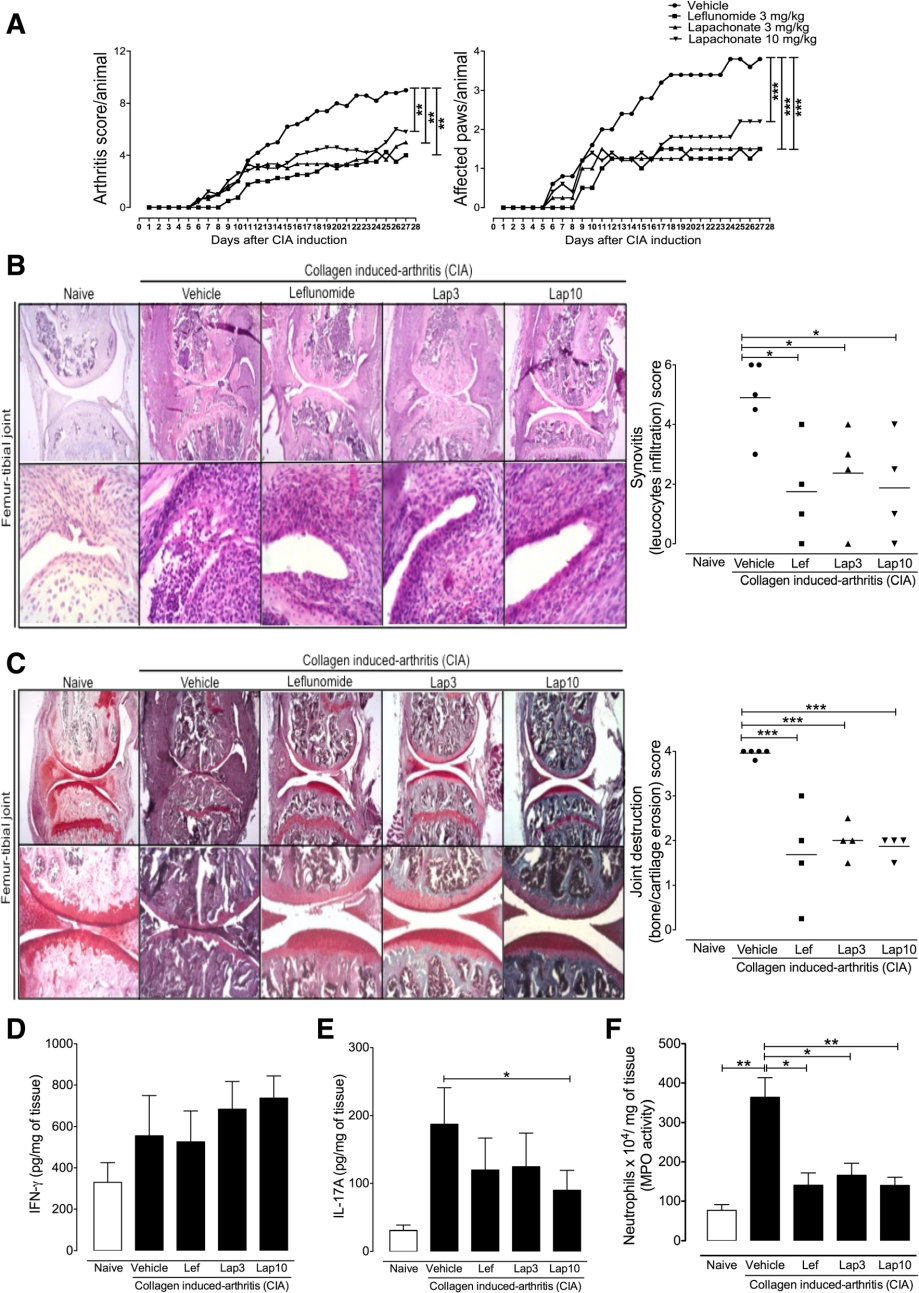
Immunomodulatory effects of LAP on collagen-induced arthritis (CIA). DBA1/J male mice were injected i.d. at the base of the tail with 200 μg CII emulsified in CFA on day 0. Mice were boosted i.d. with CII (200 μg emulsified in IFA) on day 21. After arthritis induction, mice were treated orally with lapachol (*Lap*) (3 mg/kg and 10 mg/kg) or leflunomide (*Lef*) (3 mg/kg) or saline daily. **a** Clinical score/mouse (*left panel*) and affected paws/mouse (*right panel*) were addressed daily after arthritis induction. Data represent mean, *n* = 5 mice per group. *P < 0.05, **P < 0.01, ***P < 0.001. **b**, **c** Histological analysis of CIA mice treated with LAP (3 mg/kg and 10 mg/kg) or LEF (3 mg/kg). Representative images of knee joint sections stained with H&E (**b**) or Safranin-O (**c**) and respective histopathological scores. Magnification for H&E: *upper row* 100×; *lower row* 400×; Safranin-O: *upper row* in 100×; *lower row* in 250×. Data represent mean, *n* = 5 in the vehicle and naive groups, *n* = 4 in Lef, Lap3 and Lap10 groups. *P < 0.05, **P < 0.01. Production of interferon gamma (*IFN-*γ) (**d**) and interleukin-17A (*IL-17A*) (**e**) tissue levels from paws of CIA mice at 4 weeks after the boost with CII. **f** Myeloperoxidase (*MPO*) activity from paws of CIA mice at 4 weeks after the boost with CII. Data represent mean ± SEM, *n* = 5 mice per group. *P < 0.05, **P < 0.01

We next examined the therapeutic potential of LAP in two experimental models of arthritis. The first model was collagen-induced arthritis (CIA), a well-established T cell-dependent preclinical model for RA [27]. Treatment with LAP was started just after booster injection with collagen on day 21 after the first immunization. Mice were orally treated with LAP (3 mg/kg and 10 mg/kg) once a day for 4 weeks. LAP was well tolerated without apparent side effects based on observations of the general symptoms of toxicity, including piloerection, diarrhea, weight loss, and prostration. We also found that treatment with LAP did not alter the serum levels of ALT or AST during the CIA protocol (Additional file 6: Figure S3), indicating that it was not hepatotoxic at the doses used. As a positive therapeutic control, mice were treated with LEF (3 mg/kg) using the same treatment schedule. The doses of LEF and LAP used in this therapeutic protocol were based on previous reports [28]. Clinical arthritis scores were recorded from booster injection (day 0) and graded on a scale of the magnitude of paw swelling, erythema, and ankylosis (as described in the Methods section). We found that LAP, at both doses used, markedly attenuated the severity of arthritis in CIA mice, similar to those observed in mice treated with LEF, as evidenced by a reduction of clinical score and the number of affected paws (Fig. 3a). Histopathological analysis of knee joint sections from vehicle-treated mice stained with H&E and Safranin-O revealed inflammatory cell infiltration, pannus formation, and cartilage loss when compared to naive mice (Fig. 3b and c). Notably, LAP markedly reduced all histopathological features of arthritis severity when compared to the vehicle-treated group. No significant differences in histopathological features were observed between mice treated with LAP and LEF (Fig. 3b and c). Additionally, we measured the levels of inflammatory cytokines and MPO activity, which indirectly reflects neutrophil infiltration, in the hind paws from CIA mice treated or not with LAP or LEF. We did not found any significant differences in IFN-γ levels among all groups (Fig. 3d). However, CIA mice treated with LAP with the dose of 10 mg/kg showed a significant reduction in IL-17A levels (Fig. 3e). Moreover, we found that mice treated with LAP or LEF showed reduced MPO activity compared to vehicle-treated CIA mice (Fig. 3e).

**Fig. 4:**
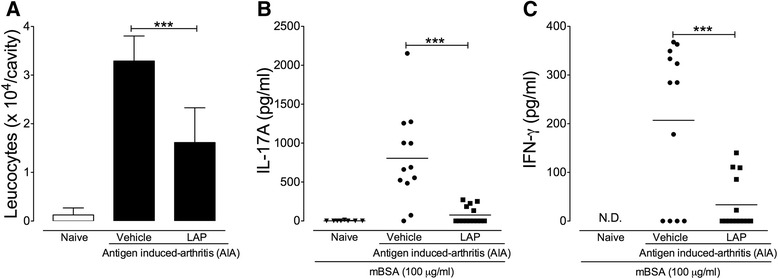
Immunomodulatory effects of LAP on antigen-induced arthritis (AIA). Methylated bovine serum albumin (*mBSA*)-immunized C57BL/6 mice were treated orally with lapachol (*LAP*) (10 mg/kg) once a day over 9 days, beginning 12 days after the first immunization. On day 21 after the first immunization, mice were challenged with an intra-articular injection of 30 μg mBSA. **a** Leucocyte infiltration into the knee joint analyzed 6 h after mBSA challenge. **b**, **c** A pool of cell suspension of draining lymph nodes (inguinal) and spleen from naive or mBSA-immunized mice treated or not with LAP stimulated with mBSA (100 μg/ml) for 96 h. Production of interleuking-17A (*IL-17A*) (**b**) and interferon gamma (*IFN-*γ) (**c**) by splenic and draining lymph node cells in response to mBSA stimulation measured by ELISA in the culture supernatant. Data represent mean ± SEM, naive (*n* = 8), vehicle (*n* = 12), and LAP (*n* = 14). ***P < 0.001. *N.D.* not determined

Finally, we investigated the immunomodulatory effects of LAP in a second model of experimental arthritis. To this end, we employed the antigen-induced arthritis (AIA) model in C57BL/6 mice, which also requires a T-cell response for the generation of the acute articular inflammation [29]. Briefly, mice were treated orally with LAP (10 mg/kg) once a day over 9 days, beginning 12 days after the first immunization with the antigen mBSA. On day 21 after the first immunization, arthritis was induced by intra-articular injection of mBSA into the knees of immunized mice. We did not find differences in the serum levels of anti-mBSA total IgG between mBSA-immunized mice treated or not (vehicle) with LAP (Additional file 7: Figure S4). However, mice treated with LAP exhibited a remarkable reduction in leucocyte infiltration into the knee joint 6 h after mBSA challenge when compared to vehicle-treated mice (Fig. 4a). We then evaluated the recall responses by cells from mBSA-immunized mice treated or not with LAP. The mBSA-specific production of IL-17 and IFN-γ by draining lymph node cells and splenocytes was significantly reduced in mice treated with LAP (Fig. 4b and c). IL-4 was not detected in the supernatant of stimulated cells (data not shown). Collectively, these findings show the marked immunomodulatory effects of LAP in two models of experimental arthritis.

## Discussion

In the present study, we conducted a series of in silico, in vitro and in vivo studies describing the biological activity and pharmacokinetic properties of LAP, which is a novel immunosuppressive drug that attenuates experimental autoimmune arthritis through inhibition of DHODH activity. Firstly, we synthetized LAP and performed chemical modifications to improve its solubility in water. In accordance with a previous report [9], we found that LAP can inhibit the enzymatic activity of hDHODH in vitro. Moreover, we also provided a convincing model for the interaction of LAP with hDHODH by computational docking studies, indicating similar interactions observed with A771726, the active metabolite of LEF. Specifically, the narrow and relatively good hydrophobic pocket of hDHODH allows a suitable accommodation of hydrophobic prenyl and aromatic moieties from LAP. In this case, the analyses predicted a consensual binding mode amongst all the poses calculated for LAP, which additionally interacts by hydrogen bonds with Arg136 and Tyr356 of hDHODH, residues well conserved amongst the mammalian enzymes [5].

LAP is a naturally occurring naphthoquinone that has been reported to exhibit antitumor, anti-inflammatory, and antimicrobial activities, but the molecular mechanism underlining these effects is poorly understood [9–15]. It was previously reported that some naphthoquinones derivatives, including LAP, can inhibit DHODH activity [9], but the biological relevance of this observation was not investigated. DHODH is a mitochondrial enzyme that catalyzes the rate-limiting step of the de novo pyrimidine synthesis [5]. Using lymphocyte proliferation assays, we demonstrated that LAP has a potent immunosuppressive activity on human and murine lymphocytes. Supplementation with uridine, which overcomes the inhibition of pyrimidine synthesis, reversed the antiproliferative activity of LAP on lymphocytes in vitro, demonstrating that the molecular mechanism underlying the antiproliferative effect is mainly due to DHODH inhibition. Importantly, we found that LAP exhibits a greater ability to suppress the proliferation of T cells than observed with LEF in vitro. These results suggest that LAP has immunosuppressive activity on lymphocytes through its direct ability to block DHODH activity and, consequently, inhibit pyrimidine synthesis.

In the pathogenesis of RA, it is well accepted that the influx and proliferation of T cells in the synovial space play a critical role in the articular inflammation and joint destruction [1, 27, 30]. In fact, autoreactive activated T cells in the joint stimulate plasma cells, mast cells, macrophages, and synovial fibroblasts to produce inflammatory mediators, which in turn stimulate matrix degradation [4]. Therefore, compounds that inhibit T-cell proliferation have been introduced into the therapeutic schedule of RA [2]. LEF is a widely used antiproliferative and immunosuppressive drug for treatment of RA that targets DHODH [4]. However, around 30–40% of RA patients do not have an appropriate response to LEF [7]. Thus, identification of new small molecule inhibitors targeting DHODH constitutes an attractive therapeutic approach for RA. Taking into account that LAP shows a great ability to inhibit DHODH in vitro, we hypothesized that LAP could have a therapeutic potential in the context of arthritis by interfering with T-cell proliferation. In accordance with its immunosuppressive activity in vitro, we found that LAP effectively attenuated arthritis development and progression in two well-established T cell-dependent models of autoimmune arthritis. Moreover, mice treated with LAP showed a reduction in joint inflammation and articular damage at similar effectiveness as LEF.

Synovial tissue infiltrating inflammatory cells from RA patients are more resistant to apoptotic events, contributing to their accumulation and, consequently, the persistence of inflammation [31]. The exact mechanism that drives the leucocyte resistance to apoptosis in RA remains unclear, but it is believed that proinflammatory cytokines released in the synovial fluid microenvironment are responsible for this phenomenon [32]. Since LAP is reducing the production of T cell-dependent proinflammatory cytokines in vivo, it could be indirectly interfering with the apoptosis of inflammatory cells. Thus, LAP and its derivate comprise a potential option for the development of novel lead candidates for treating RA based on DHODH inhibition. Indeed, β-lapachone, a closely related secondary metabolite of LAP, is a promising drug candidate currently in Phase II clinical trials for the treatment of cancer based on its ability to inhibit DHODH (ClinicalTrials.gov identifier: nCT01502800; ClinicalTrials.gov identifier: nCT02514031). However, further studies are needed to determine whether LAP will be effective in inhibiting proliferation of T cells from RA patients who show an inadequate response to LEF.

## Conclusions

In summary, this study demonstrated that LAP is a novel immunosuppressive drug that attenuates experimental autoimmune arthritis through inhibition of DHODH activity. Therefore, LAP could be considered as a potential immunosuppressive lead candidate with potential therapeutic implications for RA.

## Additional files

Additional file 1: Figure S1. Mean concentration–time profiles of lapachol after (A) 2 mg/kg i.v., (B) 10 mg/kg oral, and (C) 25 mg/kg oral administration to rats. Data points are mean ± standard deviation. (PDF 146 kb)
Additional file 2: TableS1. LAP and LAP sodium salt pharmacokinetic parameters determined by noncompartmental and compartmental approaches after i.v. administration of 2 mg/kg in Wistar rats. (PDF 93 kb)
Additional file 3: Table S2. Pharmacokinetic parameters after oral administration of lapachol (10 mg/kg and 25 mg/kg) and LAP sodium salt (30 mg/kg) in Wistar rats. (PDF 79 kb)
Additional file 4: Figure S2. Superimposition of the crystallographic hDHODH inhibitor A771726 (PDB id:1D3H, carbon atoms in cyan) and the top-ranked docking solution (carbon atoms in yellow), inside the active site. (PDF 458 kb)
Additional file 5: Table S3. Evaluation of the toxicity of lapachol in proliferating CD4 T cells. (PDF 224 kb)
Additional file 6: Figure S3. Serum levels of GPT and AST in LAP-treated mice during CIA protocol. (PDF 518 kb)
Additional file 7: Figure S4. Serum titers of anti-mBSA antibodies in LAP-treated mice on antigen-induced arthritis (AIA). (PDF 767 kb)

## Abbreviations

AIA: Antigen-induced arthritis
ALT: Alanine aminotransferase
AST: Aspartate aminotransferase
AUC: Area under the curve
CFA: Complete Freund’s adjuvant
CIA: Collagen-induced arthritis
CII: Type II collagen
Cmax: Peak plasma concentration
DCIP: 2,6-Dichloroindophenol
DHODH: Dihydroorotate dehydrogenase
DMARD: Disease-modifying antirheumatic drug
DMSO: Dimethyl sulfoxide
EDTA: Ethylenediaminetetraacetic acid
ELISA: Enzyme-linked immunosorbent assay
H&E: Hematoxylin and eosin
hDHODH: Human dihydroorotate dehydrogenase
i.v.: Intravenous
IC_50_: Half maximal inhibitory concentration
IFA: Incomplete Freund’s adjuvant
IFN: Interferon
IL: Interleukin
LAP: Lapachol
LEF: Leflunomide
mBSA: Methylated bovine serum albumin
MPO: Myeloperoxidase
MRT: Mean residence time
MSC: Model selection criterion
NCA: Noncompartmental approach
PBMC: Peripheral blood mononuclear cell
RA: Rheumatoid arthritis
t1/2: Half-life
ULPC-MS/MS: Ultra-performance liquid chromatography tandem mass spectrometry
Vdss: Volume of distribution
