# Supplementary material for: Lapachol, a compound targeting pyrimidine metabolism, ameliorates experimental autoimmune arthritis
Source: Arthritis Res Ther. 2017 Mar 7;19:47. doi: 10.1186/s13075-017-1236-x (PMC5341405; doi:10.1186/s13075-017-1236-x)
Supplement: Additional file 1: Figure S1. — Mean concentration–time profiles of lapachol after (A) 2 mg/kg i.v., (B) 10 mg/kg oral, and (C) 25 mg/kg oral administration to rats. Data points are mean ± standard deviation. (PDF 146 kb) [file 13075_2017_1236_MOESM1_ESM.pdf]

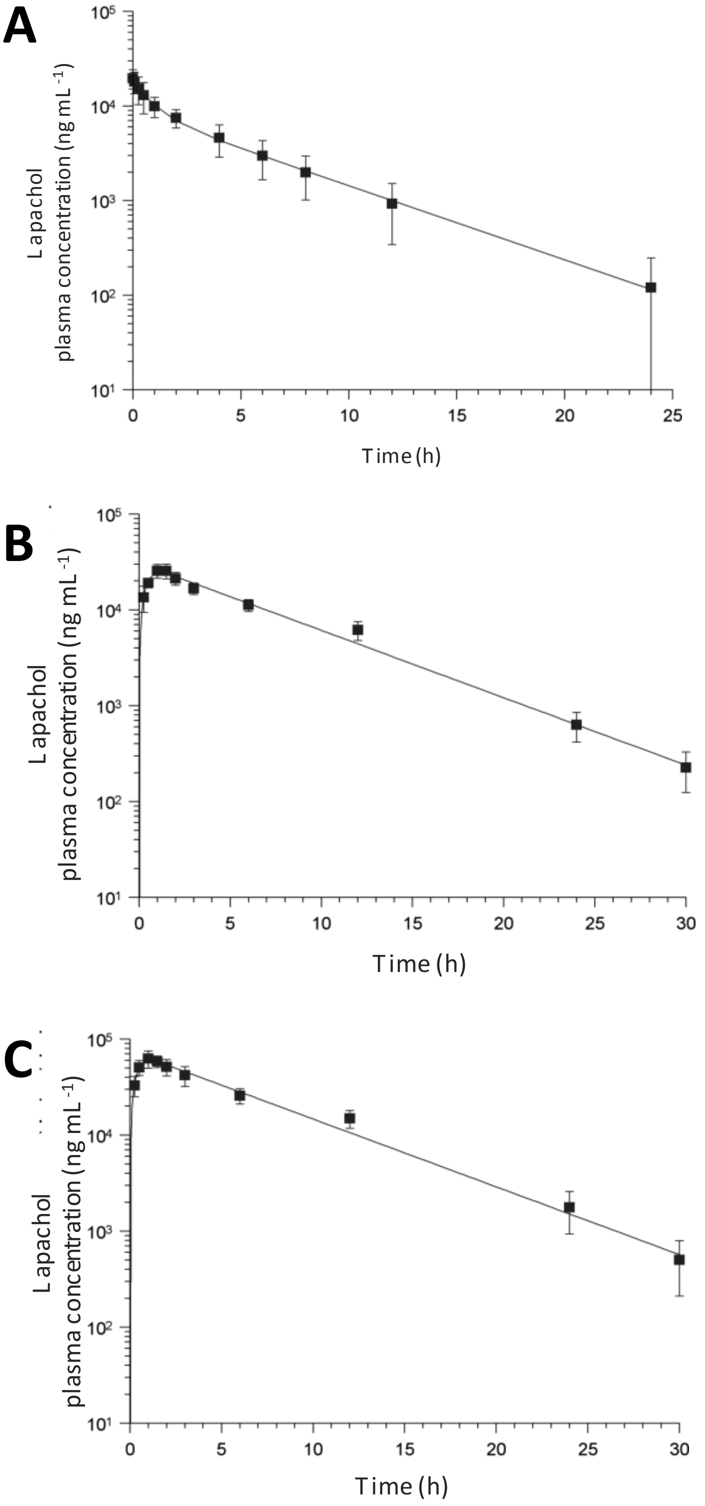

Fig. S1. Mean Concentration-time profiles of lapachol after (A) 2 mg/kg i.v., (B) 10 mg/kg oral and (C) 25 mg/kg oral administration to rats. Data points are mean ± standard deviation.

.
