# Supplementary material for: Lapachol, a compound targeting pyrimidine metabolism, ameliorates experimental autoimmune arthritis
Source: Arthritis Res Ther. 2017 Mar 7;19:47. doi: 10.1186/s13075-017-1236-x (PMC5341405; doi:10.1186/s13075-017-1236-x)
Supplement: Additional file 2: TableS1. — LAP and LAP sodium salt pharmacokinetic parameters determined by noncompartmental and compartmental approaches after i.v. administration of 2 mg/kg in Wistar rats. (PDF 93 kb) [file 13075_2017_1236_MOESM2_ESM.pdf]

Table S1. LAP and LAP sodium salt pharmacokinetic parameters determined by non-compartmental and compartmental approaches after i.v. administration of 2 mg/kg in Wistar rats

Pharmacokinetic Parameters	LAP		LAP sodium salt	
	NCA	2-Comp	NCA	2-Comp
a (μg/mL)	-	10.0 ± 4.1	-	11.0 ± 14.7
b (μg/mL)	-	8.8 ± 3.1	-	9.8 ± 1.9
α (h^{-1})	-	1.76 ± 1.52	-	8.6 ± 6.8
β (h^{-1})	-	0.19 ± 0.03	-	0.20 ± 0.03
λ (h^{-1})	0.18 ± 0.04	-	0.30 ± 0.11	
$t_{1/2}\alpha$ (h)	-	0.6 ± 0.4	-	0.19 ± 0.16
$t_{1/2}\beta$ (h)	-	3.7 ± 0.5	-	3.5 ± 0.5
$t_{1/2}$ (h)	4.1 ± 1.1	-	2.5 ± 0.8	-
$AUC_{0-\infty}$ (μg h/mL)	56.1 ± 20.1	56.4 ± 21.6	54.5 ± 26.8	50.8 ± 11.8
CL_{tot} (L/kg)	0.04 ± 0.01	0.04 ± 0.01	0.04 ± 0.02	0.04 ± 0.01
Vc (L/kg)	-	0.11 ± 0.03	-	0.12 ± 0.06
Vd_{ss} (L/kg)	0.19 ± 0.03	0.17 ± 0.03	0.29 ± 0.17	0.20 ± 0.04
MRT (h)	5.3 ± 1.2	-	6.5 ± 4.1	4.9 ± 0.8

LAP sodium salt (n =6); LAP (n = 7).

.
