# Supplementary material for: Lapachol, a compound targeting pyrimidine metabolism, ameliorates experimental autoimmune arthritis
Source: Arthritis Res Ther. 2017 Mar 7;19:47. doi: 10.1186/s13075-017-1236-x (PMC5341405; doi:10.1186/s13075-017-1236-x)
Supplement: Additional file 3: Table S2. — Pharmacokinetic parameters after oral administration of lapachol (10 mg/kg and 25 mg/kg) and LAP sodium salt (30 mg/kg) in Wistar rats. (PDF 79 kb) [file 13075_2017_1236_MOESM3_ESM.pdf]

Table S2. Pharmacokinetic parameters after oral administration of lapachol (10 mg/kg and 25 mg/kg) and LAP sodium salt (30 mg/kg) in Wistar rats

Pharmacokinetic Parameter	10 mg/kg oral LAP		25 mg/kg oral LAP		30 mg/kg oral LAP sodium salt	
	NCA	1-Comp	NCA	1-Comp	NCA	1- Comp
C_{max} (µg/mL)	31.6 ± 3.8	-	65.6 ± 10.6	-	55.9 ± 25.8	-
t_{max} (h)	1.2 ± 0.3	-	1.3 ± 0.4	-	1.6 ± 1.1	-
Ka (h^{-1})	-	2.61 ± 0.85	-	2.93 ± 0.79	-	1.04 ± 1.02
Ke (h^{-1})	-	0.17 ± 0.01	-	0.16 ± 0.02	-	0.25 ± 0.15
l (h^{-1})	0.16 ± 0.01	-	0.19±0.03	-	0.19 ± 0.07	-
$t_{1/2}$ (h)	3.2 ± 0.2	-	3.6±0.5	-	4.3 ± 2.0	-
$t_{1/2}\beta$ (h)	-	4.2 ± 0.3	-	4.4 ± 0.6	-	3.6 ± 2.0
$AUC_{0-\infty}$ (μg h/ml)	216.9 ± 22.0	237.4 ± 20.2	449 ± 78.1	434.3 ± 79.7	315.2 ± 140	314 ± 143.8
CL_{tot} (L/kg)	0.04 ± 0.01	0.03 ± 0.03	0.04 ± 0.01	0.04 ± 0.01	0.04 ±0.02	0.05 ± 0.03
Vd_{ss} (L/kg)	-	0.19 ± 0.02	-	0.24 ± 0.04	-	0.20 ± 0.09
MRT (h)	7.2 ± 0.6	-	7.2 ±0.7	-	6.5 ± 2.8	-
F_{abs} (%)	77	-	65	-	42	-

LAP (n = 8, 10 mg/kg); LAP (n = 6, 25 mg/kg); LAP sodium salt (n = 8, 30 mg/kg)
