# Supplementary material for: Lapachol, a compound targeting pyrimidine metabolism, ameliorates experimental autoimmune arthritis
Source: Arthritis Res Ther. 2017 Mar 7;19:47. doi: 10.1186/s13075-017-1236-x (PMC5341405; doi:10.1186/s13075-017-1236-x)
Supplement: Additional file 4: Figure S2. — Superimposition of the crystallographic hDHODH inhibitor A771726 (PDB id:1D3H, carbon atoms in cyan) and the top-ranked docking solution (carbon atoms in yellow), inside the active site. (PDF 458 kb) [file 13075_2017_1236_MOESM4_ESM.pdf]

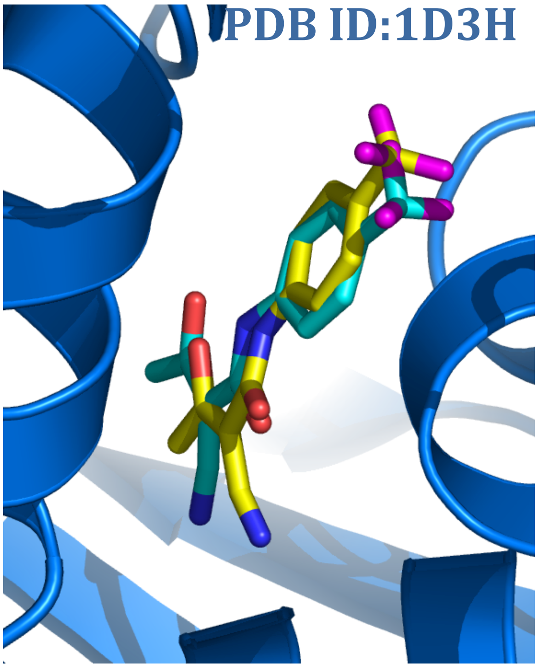

Fig. S2. Superimposition of the crystallographic hDHODH inhibitor A771726 (PDB id:1D3H, carbon atoms in cyan) and the top-ranked docking solution (carbon atoms in yellow), inside the active site.
