# Supplementary material for: Lapachol, a compound targeting pyrimidine metabolism, ameliorates experimental autoimmune arthritis
Source: Arthritis Res Ther. 2017 Mar 7;19:47. doi: 10.1186/s13075-017-1236-x (PMC5341405; doi:10.1186/s13075-017-1236-x)
Supplement: Additional file 5: Table S3. — Evaluation of the toxicity of lapachol in proliferating CD4 T cells. (PDF 224 kb) [file 13075_2017_1236_MOESM5_ESM.pdf]

Table S3. Evaluation of lapachol´s toxicity in proliferating CD4 T cells

Groups	Purified CD4 T cells Annexin V^{+} (%)	Purified CD4 T cells Annexin $V^{+}PI^{+}$ (%)
Medium	13,31 ± 0,82	5,31 ±1,71
Only anti-CD3	10,54 ± 0,68	3,70 ± 0,14
Leflunomide (10 µM)	11,08 ± 0,71	3,90 ± 0,47
Leflunomide (30 µM)	10,28 ± 0,64	3,84 ± 0,54
Leflunomide (100 µM)	10,14 ± 0,27	3,65 ± 0,71
Lapachol (10 µM)	12,99 ± 0,47	4,19 ± 0,53
Lapachol (30 µM)	12,04 ± 1,42	5,60 ± 1,00
Lapachol (100 µM)	47,38 ±10,53*	27,58 ± 6,70*

* P <0,05; analysis of variance one-way nonparametric (ANOVA) followed by Bonferroni's t test
