# Supplementary material for: Lapachol, a compound targeting pyrimidine metabolism, ameliorates experimental autoimmune arthritis
Source: Arthritis Res Ther. 2017 Mar 7;19:47. doi: 10.1186/s13075-017-1236-x (PMC5341405; doi:10.1186/s13075-017-1236-x)
Supplement: Additional file 6: Figure S3. — Serum levels of GPT and AST in LAP-treated mice during CIA protocol. (PDF 518 kb) [file 13075_2017_1236_MOESM6_ESM.pdf]

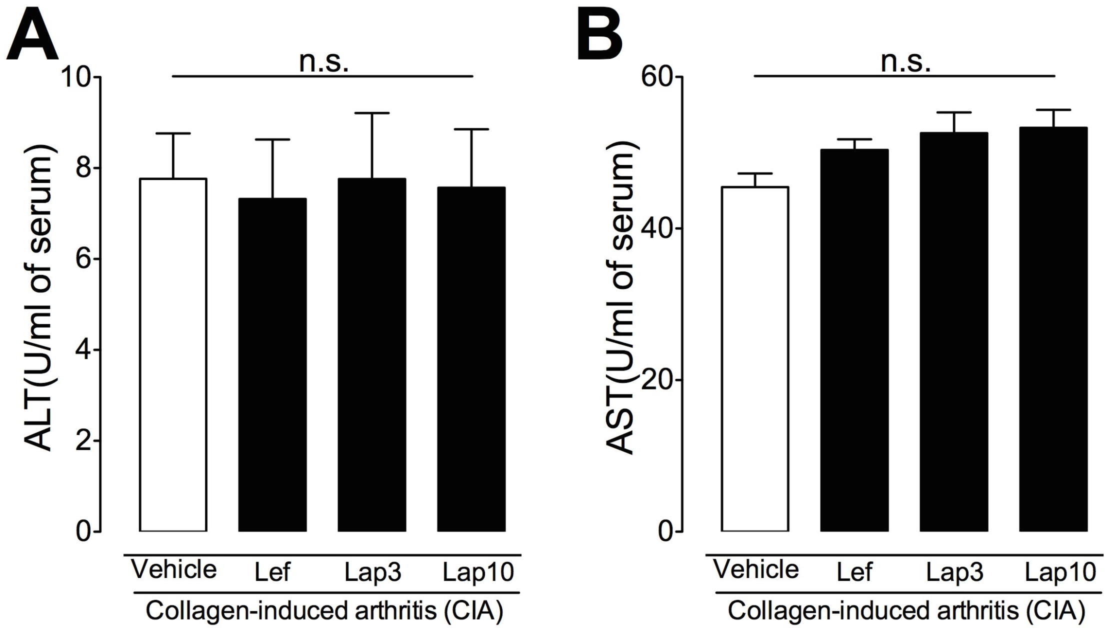

Fig. S3. Serum levels of GPT and GOT from LAP-treated mice during CIA protocol. DBA1/J male mice were injected i.d. at the base of the tail with 200 μg of CII emulsified in CFA on day 0. Mice were boosted i.d. with CII (200 μg emulsified in IFA) on day 21. After arthritis induction, mice were treated orally with LAP (3 mg/kg and 10 mg/kg) or LEF (3 mg/kg) or saline daily. (A-B) Levels of serum ALT (A) and AST (B) in CIA mice 4 wk after boost with CII. Data points are mean ± standard deviation. n.s., not significant.

.
