# Supplementary material for: Lapachol, a compound targeting pyrimidine metabolism, ameliorates experimental autoimmune arthritis
Source: Arthritis Res Ther. 2017 Mar 7;19:47. doi: 10.1186/s13075-017-1236-x (PMC5341405; doi:10.1186/s13075-017-1236-x)
Supplement: Additional file 7: Figure S4. — Serum titers of anti-mBSA antibodies in LAP-treated mice on antigen-induced arthritis (AIA). (PDF 767 kb) [file 13075_2017_1236_MOESM7_ESM.pdf]

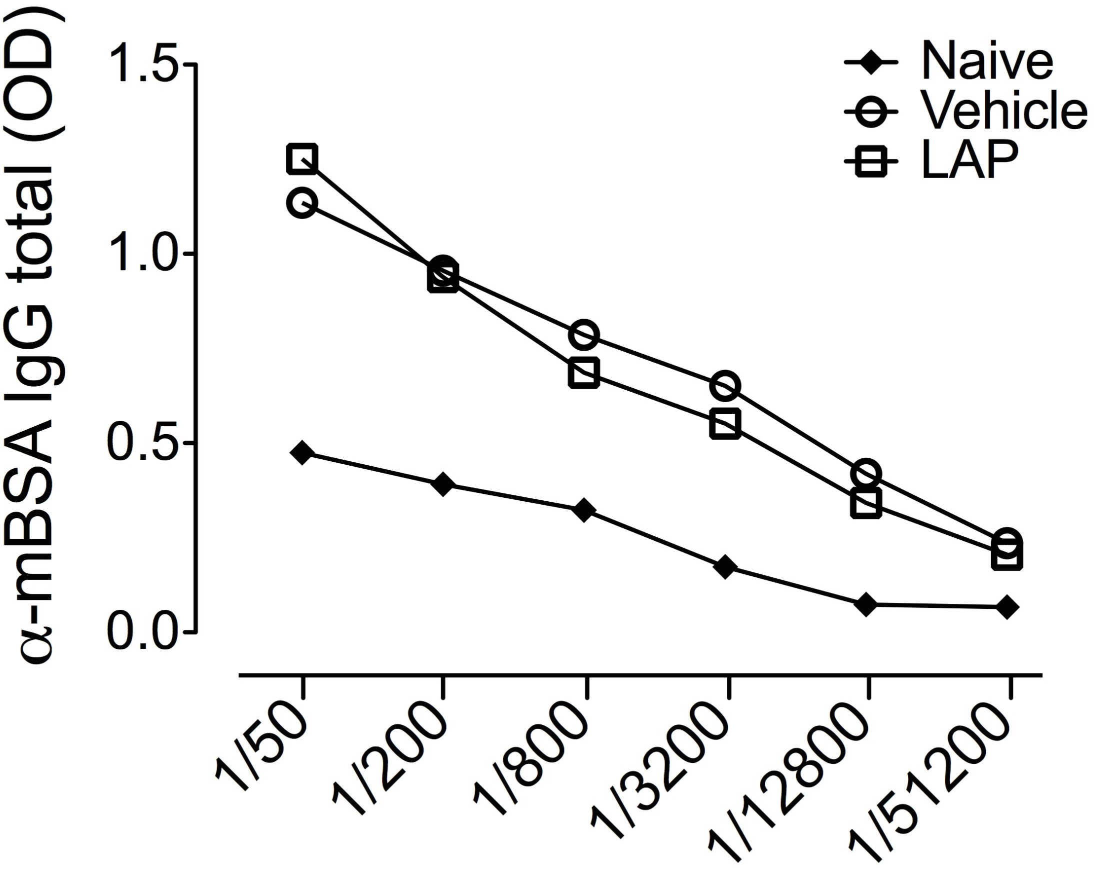

Fig. S4. Serum titers of anti-mBSA antibodies from LAP-treated mice on antigen-induced-arthritis (AIA). mBSA-immunized C57BL/6 mice were treated orally with LAP (10 mg/kg) or vehicle every day from 12-21 day after first immunization. The graph shows the anti-mBSA IgG levels in the serum from naive and mBSA-immunized mice treated or not with LAP on day 21.

.
